# Tincture of Iodine Removes Corus

**Published:** 1860-05

**Authors:** 


Tincture of Iodine removes Corns.—By means of a camel's hair brush, wet the corn several times a day for a short time; the pain gradually disappears, the skin becomes thinner, acquiring, finally, its original softness. To prevent a return, all pressure of the shoe must be avoided.—*Zeitschrift d. Pharmabie, Leipzig*, 1859.

